# Pre-operative Intraperitoneal Drainage Under Local Anesthesia: A Promising Resuscitation Tool in Peritonitis Secondary to Hollow Viscus Perforation During the Corona Pandemic

**DOI:** 10.7759/cureus.15083

**Published:** 2021-05-18

**Authors:** Talha Kareem, Junaid Hashmi, Farrukh Aftab, Muhammad Ali Rabbani, Natasha Saleem, Syed Muhammad Ali

**Affiliations:** 1 General Surgery, Nishter Medical University and Hospital, Multan, PAK; 2 Surgery, Nishter Medical University and Hospital, Multan, PAK; 3 Anatomy, Combined Medical Hospital Institute of Medical Sciences, Multan, PAK; 4 Acute Care Surgery, Hamad General Hospital, Doha, QAT; 5 Surgery, Weill-Cornell Medicine Qatar, Doha, QAT

**Keywords:** perforation peritonitis, intraperitoneal drains, laparotomy, in-hospital mortality, coronavirus

## Abstract

Introduction

To study the role of intraperitoneal drainage in the resuscitation of patients with perforation peritonitis during the corona pandemic.

Materials and methods

This retrospective study was conducted in the general surgery department of Nishtar Medical University/Hospital Multan from April 2020 to September 2020. Patients of peritonitis who presented with bilateral pulmonary crepitations, SpO2 less than 92%, PaO2 less than 60 mmHg on arterial blood gas (ABG), and chest x-ray (CXR) finding of bilateral infiltrates are included. Due to high suspicion of associated coronavirus infection such patients needed polymerase chain reaction (PCR) for virus detection and special measures were required for resuscitation before any definitive treatment. A delay of six to eight hours is usually encountered while ensuring proper safety measures and dedicated operation theaters. Initial resuscitation started with intravenous fluids and broad-spectrum antibiotics were given to all patients. Twenty-seven patients were resuscitated with preoperative intraperitoneal drainage while waiting for PCR and 13 patients were resuscitated without drainage. Data analysis was carried out using the statistical package for the social sciences (SPSS) version 19 software. The mean was calculated for age while frequency and percentages were calculated for gender, comorbidities, and causes of delay. The mortality was compared using the chi-square test.

Results

The mean age of patients was 43.73 ± 16.04 years. The common cause of peritonitis were perforations due to duodenal ulcer, typhoid, tuberculous (TB), and biliary origin. The variables that led to suspicion of coronavirus were SpO2 < 92%, PaO2 <60mmHg and bilateral infiltrates on chest x-ray. PCR for coronavirus was positive in nine patients. Mortality was 29.6% in those resuscitated with intraperitoneal drainage before the definitive procedure and 54% in those not resuscitated with intraperitoneal drainage.

Conclusions

The surge of coronavirus infection has put the healthcare staff at great risk. This has led to strict protocols and precautionary measures in the management of patients with perforation peritonitis with suspected corona infection. The local guidelines for the management of patients with acute abdomen should include aggressive measures right from the start during the corona pandemic. Intraperitoneal drainage, early in the management of perforation peritonitis decreases morbidity and mortality in suspected corona infected patients.

## Introduction

Since the beginning of 2020, there have been major changes in the management of patients in hospitals due to the corona pandemic. The safety of healthcare staff has been a major concern worldwide. Although the management of critical patients has not changed, the provision of all precautionary measures has been a serious concern worldwide [1]. Peritonitis is one of the common presentations of general surgery emergency departments around the globe and is associated with high mortality rates. The presence of the SARS CoV-19 virus in blood and feces requires the safe handling of patients with acute abdomen in the emergency department [2,3]. Proximal gastrointestinal perforations are more common as compared to distal perforations [4,5].

For decades, there have been ongoing developments to develop the best management option for peritonitis. In the 19th century, conservative (non-surgery) management was the standard management. In the late 19th century, surgical management developed, and medical management became disputed because of high morbidity and mortality [6].

The mortality rate of peritonitis has been reported to be 6% to 27% [7,8]. The reported risk factors of high mortality are: old age, co-morbidities, late presentation, and delay in starting treatment [9]. Therefore, early diagnosis and prompt management are mainstays of favorable outcomes [10]. Early aggressive surgical management is the definitive option to control contamination and bacterial load to prevent septicemia.

Front line health care workers including surgical teams of emergency departments are one of the most vulnerable hospital staff in the pandemic. The detection of SARS CoV-19 in peritoneal fluid implies that all the suspected patients should be treated as positive until proven otherwise [11]. Although, treatment of the suspected patient cannot be deferred, a conservative approach initially is being used to ensure proper safety measures like the provision of personal protective equipment, for hospital staff and arranging operation rooms specific for such patients. Despite huge advancements in surgical techniques, antibiotics, and intensive care management the morbidity and mortality are still very high in these patients secondary to cytokine storm and lack of specific antiviral treatment at the beginning of the pandemic. Therefore, different techniques like aggressive fluid resuscitation, the commencement of broad-spectrum antibiotics, and early surgical intervention are still being investigated to improve outcomes of peritonitis. One of these is the use of pre-operative drains to reduce the burden of contamination and improved survival [12]. The present study is designed to evaluate the outcomes of pre-op insertion of intra-peritoneal drains under local anesthesia before surgical intervention in patients with peritonitis.

## Materials and methods

This retrospective study was conducted in the general surgery department of Nishtar Medical University/Hospital Multan, Pakistan from April to September 2020. Patients with peritonitis who presented with bilateral pulmonary crepitations, SpO2 less than 92%, PaO2 less than 60 mmHg on arterial blood gas (ABG), and chest x-ray (CXR) finding of bilateral infiltrates, were included in the study. Due to the suspicion of associated coronavirus infection, a polymerase chain reaction (PCR) test was sent for all these patients before any definitive surgical treatment which resulted in a delay of six to eight hours while taking proper measures to deal with corona suspected patients.

Initial resuscitation using standard hospital protocols was started in each patient with intravenous fluids, broad-spectrum antibiotics, and proper analgesia. Intraperitoneal drainage during resuscitation was carried out in patients with gross abdominal distention, respiratory distress, and septic shock with proper personal protective equipment (PPE). During resuscitation, the vital signs of patients were monitored to assess maintaining stability.

Drains were inserted by making a 2-2.5 cm skin incision on either flank under local anesthesia (LA). The choice of flank was made on clinical assessment and location of collection on ultrasonography. The abdominal muscles were split using artery forceps. After abdominal access, the finger was inserted into the cavity and the muscles were wrapped in all directions for protection during drain insertion and to allow uniform drainage. Two drains of size 28-32 Fr were inserted in each patient after exploration. The direction of one drain was kept upright and for the second towards the pelvic cavity. Drained fluid was sent for culture and sensitivity only and not for COVID PCR.

A nasogastric tube and foley catheter was inserted in every patient and vital signs and urine output were monitored. Fluid resuscitation and empirical broad-spectrum antibiotics covering gram-negative and positive bacteria along with anaerobes were started for all patients. However, after culture reports, antibiotics sensitive to the specific strains were started. Drain output and vital signs were noted immediately and after every two hours. Abdominal ultrasonography (USG) was done after six hours to monitor the level of fluid in the peritoneal cavity and all patients were operated on within eight hours of presentation. Parenteral nutrition was started if the patient was kept nil per oral (NPO) for >5 days after starting the treatment.

## Results

A total of 40 patients were included in this study. The mean age of patients was 43.73 ± 16.04 years, with an 85% male population. The most common co-morbid conditions were chronic obstructive airway disease (COPD) diagnosed in 50% of patients, ischemic heart disease in 35%, diabetes mellitus in 35%, and hypertension in 35% of patients (Table 1). Out of the 27 patients who were resuscitated with intraperitoneal drainage during pre-operative care, 19 were treated successfully and eight expired during the post-operative period. On the other hand, out of 13 patients who were resuscitated without drainage procedure six were discharged, and the remaining expired.

**Table 1:** Baseline Characteristics of Patients. Age = 43.73 ± 16.04 years

Variable	N	%age
Age ≤ 50 years	24	60.0%
> 50 years	16	40.0%
Gender Male	34	85.0%
Female	6	15.0%
Comorbidities Hypertension	14	35.0%
Diabetes Mellitus	14	35.5%
Ischemic Heart Disease	14	35.5%
COPD	20	50.0%
Asthma	6	15.0%

The common cause of peritonitis was duodenal ulcer perforation, typhoid perforation, tuberculous perforation, and biliary peritonitis. The variables that led to suspicion of coronavirus were SpO2 < 92%, PaO2 <60mmHg and bilateral infiltrates on chest x-ray and covid testing was done on all of these patients. Delay in definitive treatment was six to eight hours. Mortality was 29.6% in those resuscitated with intraperitoneal drainage before the definitive procedure and 54% in those in whom intraperitoneal drainage was not carried out (Table 2).

**Table 2:** Results of Pre-Operative Intraperitoneal Drainage

Manage categories	n	Percentage treated	Mortality
Drainage Pre-operatively	27	70%	29.6%
Drainage not done Pre-operatively	13	46%	54%

PCR for coronavirus was positive in nine patients (out of 40) and mortality was 66.7% due to respiratory complications and post-operative intra-abdominal sepsis (Table 3).

**Table 3:** Mortality Among Covid Positive Patients

Covid positive patients n	Treated	Mortality
9	3 (33.3%)	6 (66.7%)

## Discussion

Management of patients in the emergency department has become more challenging during the corona pandemic. Personal safety and timely decisions regarding patient management have acquired a more central role. Newer guidelines from all over the world insist on using dedicated theatres for these patients. These theatres must have separate corridors and the number of medical staff should be the minimum. Laparoscopic procedures and the use of electrosurgical devices in the emergency department should also be kept to a minimum [13]. Gloves, goggles, face shields, and gowns have a primary role in personal protection. The primary goal of peritonitis management is to prevent infection by using anti-microbial agents and to provide adequate fluid resuscitation for hemodynamic stability because peritonitis is associated with significant fluid sequestration and hypovolemia, which is further exacerbated by vomiting and occasionally diarrhea. Recent guidelines agree on the initial management of peritonitis using fluid resuscitation and the immediate start of empirical antimicrobials [14-16]. The goal of fluid resuscitation is to maintain central venous pressure 8-12 mmHg, mean arterial pressure ≥65 mmHg, and urine output minimum of 0.5 mL/Kg/hour [17].

Keeping in view the higher mortality rate associated with delay in surgical intervention intra-peritoneal drain insertion was developed to remove debris and toxic fluid from the peritoneal cavity to reduce the bacterial and toxin load. Kim et al. first reported the use of drains in children with necrotizing enterocolitis perforation. They reported that drain insertion is beneficial for the management of perforations in children with a 56.0% success rate [18]. In the present study, there were more male patients (85%) who presented with perforated peritonitis, and a study by Baloch et al. [19] reported a male proportion of 58% in patients with severe peritonitis while a study by Afridi et al. reported a male proportion of 68.3% in peritonitis patients [20].

In our study, the most common source of peritonitis was duodenal ulcer perforation, which was diagnosed in 50% of patients, followed by biliary peritonitis after cholecystectomy and enteric fever. A study by Chakma et al. reported duodenal ulcer perforation in 54.29% of patients, typhoid ulcers in 21.43%, appendicular in 11.22% patients, and traumatic perforation in 8.57% of patients [21]. Another study by Yadav et al. reported duodenal ulcer perforation in 26.4% patients, typhoid perforation in 26.4% patients, tuberculosis in 10.3% patients, and stomach perforation in 9.2% of patients [22].

In the present study, mortality occurred in 29.6% of patients with peritonitis in whom resuscitation included intraperitoneal drainage. A study by Bhasin et al. reported a series of 60 critically ill patients of peritonitis in whom intra-peritoneal drains were inserted. They reported a mortality rate of 66.67%, which is very high as compared to ours [23]. A study by Baloch et al. reported a mortality rate of 16% that is comparable to the present results. The mortality in patients with peritonitis is influenced by many factors which may include; cause of perforation and associated morbidities [19]. A study by Veliyev found a high mortality rate in patients having peritonitis due to duodenal ulcer perforation as compared to those with gastric ulcer perforation [24]. In the present study, we did not find any association of etiology of perforation with mortality rate. However, we found a significant association of co-morbidities with mortality rate. Ischemic heart disease, diabetes, and hypertension, and COPD were the main comorbidities associated with increased mortality. 

## Conclusions

During the corona pandemic insertion of intraperitoneal drains is beneficial in terms of reduced mortality in patients with perforation peritonitis when strict protocols for appropriate safety measures are required to buy some time and stabilize the patients. The surge of coronavirus infection has placed the healthcare staff at great risk of acquiring infection. This has led to an initial conservative approach in patients of perforation peritonitis with suspected corona infection while safety measures and dedicated theatres are being arranged. The local guidelines of emergency department management of patients with acute abdomen should include aggressive measures right from the start. Intraperitoneal drainage early in the management of perforation peritonitis decreases morbidity and mortality in suspected corona-infected patients and the recent introduction of rapid antigen testing can reduce the delay to take the patients as soon as possible for definitive surgical treatment.
